# Cholesterol Sulfate Exerts Protective Effect on Pancreatic β-Cells by Regulating β-Cell Mass and Insulin Secretion

**DOI:** 10.3389/fphar.2022.840406

**Published:** 2022-03-04

**Authors:** Xueping Zhang, Dan Deng, Daxin Cui, Yin Liu, Siyuan He, Hongmei Zhang, Yaorui Xie, Xiaoqian Yu, Shanshan Yang, Yulong Chen, Zhiguang Su

**Affiliations:** ^1^ Molecular Medicine Research Center and National Clinical Research Center for Geriatrics, West China Hospital, and State Key Laboratory of Biotherapy, Sichuan University, Chengdu, China; ^2^ Department of Clinical Laboratory, Sichuan Provincial Peoples Hospital Jinniu Hospital, Chengdu, China

**Keywords:** cholesterol sulfate, pancreatic β-cells, diabetes, mitochondria, reactive oxygen species, proliferation, apoptosis

## Abstract

**Rational:** Cholesterol sulfate (CS) is the most abundant known sterol sulfate in human plasma, and it plays a significant role in the control of metabolism and inflammatory response, which contribute to the pathogenesis of insulin resistance, β-cell dysfunction and the resultant development of diabetes. However, the role of CS in β-cells and its effect on the development of diabetes remain unknown. Here, we determined the physiological function of CS in pancreatic β-cell homeostasis.

**Materials and Methods:** Blood CS levels in streptozotocin (STZ)- or high-fat diet-induced diabetic mice and patients with type 1 or 2 diabetes were determined by LC-MS/MS. The impact of CS on β-cell mass and insulin secretion was investigated *in vitro* in isolated mouse islets and the β-cell line INS-1 and *in vivo* in STZ-induced diabetic mice. The molecular mechanism of CS was explored by viability assay, EdU incorporation analysis, flow cytometry, intracellular Ca^2+^ influx analysis, mitochondrial membrane potential and cellular ROS assays, and metabolism assay kits.

**Results:** Plasma CS levels in mice and humans were significantly elevated under diabetic conditions. CS attenuated diabetes in a low-dose STZ-induced mouse model. Mechanistically, CS promoted β-cell proliferation and protected β-cells against apoptosis under stressful conditions, which in turn preserved β-cell mass. In addition, CS supported glucose transporter-2 (GLUT2) expression and mitochondrial integrity, which then resulted in a less reactive oxygen species (ROS) generation and an increase in ATP production, thereby enabling insulin secretion machinery in the islets to function adequately.

**Conclusion:** This study revealed a novel dual role of CS in integrating β-cell survival and cell function, suggesting that CS might offer a physiologic approach to preserve β-cells and protect against the development of diabetes mellitus.

## Introduction

Type 2 diabetes (T2D) is thought of as a progressive disease characterized by a gradual deterioration of pancreatic β-cell function and a reduction in β-cell mass, which occurs very early in the course of the disease (Chen et al., 2017; Esser et al., 2020). Recent studies demonstrate that intervention to improve metabolic control during the early stages of disease may preserve or even reverse β-cell function (Hannon et al., 2014). Therefore, pharmacological agents suitable for β-cell preservation have attracted considerable interest as therapeutic options to prevent or delay the onset of T2DM.

In addition to being a predominant sterol sulfate in the circulation with a concentration ranging from 1.3 to 2.6 μg/ml (Meng et al., 1997), cholesterol sulfate (CS) is also distributed in other biological fluids and tissues, including seminal plasma, urine, bile, skin, adrenal glands, uterine endometrium, kidney and liver (Strott and Higashi 2003). CS plays an important role in skin development, cell adhesion and dissociation, and blood clotting system homeostasis (Sanchez et al., 2021). As a hydrophilic excretion form of cholesterol, CS can modulate cholesterol homeostasis by targeting 3-hydroxy 3-methylglutaryl-CoA reductase (HMGCR), a key enzyme in the cholesterol synthesis pathway, and lecithin-cholesterol acyltransferase (LCAT) for esterification of cholesterol. Cholesterol metabolism is tightly linked to the function of pancreatic β-cells, and cellular cholesterol accumulation may impact β-cell function and contribute to the pathogenesis of diabetes (Perego et al., 2019). Recent evidence also demonstrates that CS regulates glucose metabolism and energy homeostasis, directly, by inhibiting gluconeogenesis via the suppression of hepatocyte nuclear factor 4α (HNF4α) (Shi et al., 2014) and, indirectly, acts as a putative natural agonist of nuclear receptor retinoic acid-related orphan receptor α (RORα) (Kallen et al., 2004). Indeed, the latter has been reported to be involved in the control of blood glucose levels and the occurrence of diabetes through the regulation of gluconeogenesis (Madsen et al., 2016; Zhang Xueping et al., 2018), lipogenesis (Kim et al., 2017), insulin production (Kuang et al., 2014) and insulin sensitivity (Liu et al., 2017a). Additionally, CS is found to be a modulator of inflammation and the immune response by negatively regulating several key targets, such as inflammatory mediator 5-lipoxygenase (5-LO) (Aleksandrov et al., 2006), T-cell receptor (TCR) (Wang et al., 2016), dedicator of cytokinesis 2 (DOCK2) (Sakurai et al., 2018) and macrophage inducible C-type lectin (Mincle) (Kostarnoy et al., 2017). It is becoming increasingly clear that inflammatory changes, including the accumulation of macrophages, contribute to the pathogenesis of insulin resistance, β-cell dysfunction and the resultant development of diabetes (Boni-Schnetzler and Meier 2019). Indeed, therapeutic inhibition of interleukin-1β (IL-1β) ameliorates β-cell dysfunction in humans (Sloan-Lancaster et al., 2013). Given its diverse effects on metabolism and metabolic disease, CS likely plays essential roles in the maintenance of β-cell homeostasis. However, the physiological function of CS in β-cells has not been defined.

To gain insight into the possible role of CS in pancreatic β-cell mass and function, we investigated the impact of CS on the viability and insulin secretion of β-cells *in vivo* and *in vitro*. We showed that CS prevented diabetes in an STZ-induced mouse model. Mechanistically, our studies demonstrated that CS stimulates β-cell proliferation and can protect cells from many deleterious processes under diabetic conditions. In addition, we found that CS can preserve β-cell function by supporting GLUT2 expression and cellular mitochondrial integrity. Our findings suggest the merit of further investigation of CS as a therapeutic approach to preserve β-cells in diabetes mellitus.

## Methods

### Clinical Specimens

The use of human tissue was compliant with the Declaration of Helsinki and the study protocol was approved by the Ethics Committee of the West China Hospital of Sichuan University. A total of sixteen (eight males and eight females) patients with T2D and eighteen (nine males and nine females) age-matched healthy controls, 12 male patients with type 1 diabetes (T1D) and eight male age-matched normal voluteers were recruited in this study. All participants signed an informed consent form for the use of serum for research purposes. Blood samples from each participant were collected after an overnight fast. The plasma was separated by centrifugation at room temperature.

### Animals and Treatments

All animal studies were approved by the Laboratory Animal Care and Use Committee of West China Hospital of Sichuan University. Seven-week-old male C57BL/6J mice were purchased from Huafukang Bioscience Technology Co. (Beijing, China). Mice were housed in a standard laboratory animal facility under a 12-h light-dark cycle with free access to standard chow food and water. After 1 week of acclimatization, mice were randomly divided into four groups (*n* = 10 in each group): the control group with an injection of saline vehicle DMSO (5% v/v), CS-treated group with an administration of CS (25 mg/kg body weight/dose, two doses daily) (Bide Pharmatech Ltd., Shanghai, China). CS dosage was selected from previous studies on its beneficial effect on glucose and lipid metabolism (Shi et al., 2014), and this regiment of CS was not toxic to the mice (Bi et al., 2018). STZ-treated group with a 7-day injection of saline vehicle followed by a 4-day administration of STZ (40 mg/kg of body weight, once daily) (Meilun Bio, Dalian, China), and CS + STZ-treated group with a 7-day CS injection followed by a STZ administration for four continuous days. In the control and STZ-treated groups, saline vehicle was intraperitoneally injected daily for 42 days, while the CS- and CS + STZ-treated groups were given the same volume of CS twice daily instead of saline (Supplementary Figure S1A). Mice were weighed weekly, and blood glucose levels were measured daily during the experimental treatment period. In addition, obesity was induced in ten male mice by feeding them a high-fat diet (HFD) (60% fat, TROPHIC Animal Feed High-Tech Co., Ltd., Jiangsu, China) for 8 weeks.

### Measurement of Fat Mass by Microcomputed Tomography

Anesthetized mice were laid in a supine position in an imaging cell, and three-dimensional (3D) X-ray images were acquired using a SPECT-μCT system (General Electric, Waukesha, United States). Adipose tissue was recognized by the reduced attenuation of X-rays in comparison with other structures. Total adipose tissue volume was determined between the proximal end of lumbar vertebra 1 (L1) and the distal end of caudal vertebra 4 (C4).

### Metabolic Analysis

The plasma levels of insulin, glucagon, triglyceride and cholesterol were measured as previously described (Zhang Yanjie et al., 2018). The intraperitoneal glucose tolerance test (IPGTT), glucose-stimulated insulin secretion (GSIS) and intraperitoneal insulin tolerance test (IPITT) were conducted in mice with overnight fasting, as previously described (Liu et al., 2017a).

### CS Measurement

CS levels in mouse and human plasma were determined using liquid chromatography tandem-mass spectrometry (LC-MS/MS) as previously described (Bandaru and Haughey 2014). Briefly, 30 μL of plasma was mixed with 120 μL of honokiol internal control solution (honokiol dissolved in methanol, 200 ng/ml). After centrifugation at 10,000 g for 15 min, 0.2 μL of the supernatant was injected into a Shimadzu LC-20AD HPLC. Chromatography was conducted using an Agilent Extend 5 μM C18 column (Agilent Technologies, Inc., Santa Clara CA, United States) with solvent A (pure methanol) and solvent B (0.01% ammonia) under the gradient conditions of 60% A at 0–1 min and gradient to 95% A at 1–2.2 min at a flow rate of 0.5 ml/min. Quantification of CS was performed on an AB SCIEX Triple Quad 5500 LC/MS/MS system (AB SCIEX, United States) by multiple reaction monitoring (MRM) using Analyst 1.6.2 software (Applied Biosystems). CS stock solution with a concentration range of 0.5 ∼3.0 μg/ml was used as an internal standard, and calibration curves were plotted using the peak area ratios of CS to honokiol (internal control).

### *In Vivo* Labeling With BrdU

Mice were given BrdU intraperitoneally at a dose of 50 mg/kg body weight once daily for 3 days. Two hours after the final injection, mice were euthanized and pancreata processed for histology.

### Mouse Islets Isolation and Insulin Secretion

Pancreatic islets were isolated from 10-week-old C57BL/6J mice using the intraductal collagenase digestion technique (Li et al., 2009). Isolated islets were plated on 12-well plates overnight and then incubated with DMSO or 10 μM CS for 12 h. Thereafter, islets were incubated in Krebs-Ringer bicarbonate (KRB) buffer with low glucose (2.8 mM) or high glucose (16.7 mM) for 1 h at 37°C. Insulin in islets was extracted by overnight incubation with ethanol/HCl buffer at 4°C. The insulin secreted into the buffer was measured using an ELISA kit (Crystal Chem, IL).

### Measurement of Glucose Uptake by 2-NBDG Staining

Dissociated islets (10 islets/well) or INS-1 β-cells (1×10^4^ cells/well) seeded in 96-well plates were incubated with DMSO or 10 µM CS for 12 h followed by a 30-min incubation of 100 μg/ml 2-NBDG (Cayman Chemical, MI, United States) in KRB buffer. The fluorescence was measured using a fluorescence plate reader with an excitation wavelength of 475 nm and an emission wavelength of 550 nm.

### Histology and Immunostaining

As previously described (Liu et al., 2017a; Chen et al., 2019), hematoxylin and eosin (HE) staining and immunohistochemistry (IHC) were conducted on paraffin sections, and immunofluorescence staining was performed on frozen sections of mouse pancreas with antibodies presented in Supplementary Table S2.

### Assessment of Intracellular Ca^2+^ Influx by Fluo-3 AM Staining

As previously described (Liu et al., 2021), isolated mouse islets were plated on 12-well plates overnight and then incubated with or without 10 µM CS for 12 h. Afterwards, islets were incubated in 5 µM Fluo-3 AM working solution (Fluo-3 AM dissolved in anhydrous DMSO was diluted with KRB buffer) at 37°C for 60 min. Subsequently, islets were perfused with KRB buffer-based solutions containing glucose (16.7 mM) or KCl (30 mM) at 37°C at a flow rate of 2 ml/min. Fluorescence imaging was conducted in an N-STORM and A1 confocal scanning microscope (Nikon, Japan).

### Measurement of ATP Levels

INS-1 β-cells plated on black 96-well plates were treated with or without 10 µM CS for 12 h following a 20-min incubation with glucose at a concentration of 2.8 mM or 16.7 mM. ATP levels in cell lysates were measured using a cellular ATP determination kit (Beyotime Biotech, Shanghai, China) in accordance with the manufacturer’s protocol.

### Transmission Electron Microscopy

Mice pancreas sections were postfixed in 1% OsO_4_, washed in 0.1 M phosphate buffer, and dehydrated via a graded ethanol series. Samples were embedded in propylene oxide/Polybed 812 epoxyresin overnight. Ultrathin sections (70 nm) stained with 2% uranyl acetate and 1% lead citrate were examined under a Philips CM120 scanning TEM.

### Cell Viability Analysis

INS-1 β-cells were seeded in 96-well plates (1×10^4^ cells/well) overnight and then incubated with or without varying concentrations of CS for 6 h followed by a coincubation of CS and STZ (2.5 mM), or H_2_O_2_ (200 µM) or palmitic acid (PA, 400 µM) for 12 h or high concentration glucose (HG, 33.3 mM) for 72 h. Cell viability was assessed by a Cell Counting Kit-8 (CCK-8) (4A Biotech, Beijing) assay according to the manufacturer’s instructions.

### Evaluation of Apototic Cells

Primary islet apoptosis was analyzed by means of terminal deoxynucleotidyl mediated dUTP nick end labeling (TUNEL) following the manufacturer’s instructions (Yeasen Biotech, Shanghai, China). The samples were stained with DAPI to visualize total cells. The ratios of TUNEL-positive nuclei to the total islet nuclei were calculated from the digitized images. INS-1 cell apoptosis was assessed by flow cytometric analysis (Thermo Scientific, United States) after cells were stained with Annexin V (AV)-fluorescein isothiocyanate (FITC)/propidium iodide (PI) (4A Biotech, Beijing, China). Data were analyzed using Flow Jo v.10.0.7 software (Tree Star, United States), and the number of apoptotic cells was expressed as the percentage of the total number of cells.

### 5-Ethynyl-2′-Deoxyuridine (EdU) Assay

INS-1 β-cells plated on 6-well plates (1×10^5^ cells/cm^2^) were incubated with DMSO or 10 µM CS for 12 h and then stained with EdU and Hoechst 33,342 dye sequentially. Images were captured by a laser scanning confocal microscope.

### Reactive Oxygen Species Assay

Intracellular ROS was estimated using a 2′,7′-dichlorofluorescin diacetate (DCFDA) Cellular ROS Assay Kit (Beyotime, Shanghai, China) following the manufacturer’s protocol. Briefly, INS-1 β-cells (1×10^4^ cells/well) or mouse islets (10 islets/well) were seeded in black 96-well plates. After a 6-h preincubation of CS (10 µM) or DMSO, cells were coincubated with CS (10 µM) and STZ (2.5 mM) or H_2_O_2_ (200 µM) or palmitic acid (PA, 400 µM) for an additional 12 h. Afterwards, the cells were incubated for 30 min at 37°C with 20 µM DCFHDA. The fluorescence was examined using an AxioVert fluorescence microscope (Carl Zeiss) with a 448-nm excitation filter. The fluorescence intensity was quantified by ImageJ software and normalized to the number of cells in the field. Cells treated only with DMSO or STZ were used as a negative or positive control, respectively.

### Assessement of Mitochondrial Membrane Potential

MMP was determined using a 5,5′,6,6′-tetrachloro-1,1′,3,3′-tetraethylbenzi-midazolylcarbocyanine iodide (JC-1) fluorescent probe (Beyotime Biotech, Shanghai, China). After the indicated treatments as described above, mouse islets or INS-1 β-cells were incubated with JC-1 staining solution for 30 min at 37°C and then analyzed using an Axiovert fluorescence microscope (Carl Zeiss) with 488-nm and 561-nm emission filters.

### RNA Isolation and Quantitative Real-Time PCR

Total RNA was extracted from mouse islets or INS-1 cells with TRIzol reagent (Invitrogen, Carlsbad, CA) and reversely transcribed into cDNA using a reverse-transcription kit (Invitrogen). qPCR analysis with the indicated primers (Supplementary Table S1) was carried out as described elsewhere (Liu et al., 2017b).

### Western Blotting Assay

Protein extraction from mouse islets or INS-1 cells and Western blotting with the indicated primary antibodies (Supplementary Table S2) were performed as previously described (Kuang et al., 2014).

### Statistical Analysis

The data were presented as the mean ± standard deviation (SD) from at least three independent experiments. The data were analyzed using SPSS 22.0 software (SPSS, United States). Statistical significance with *p* < 0.05 was determined by using a two-tailed Student’s t test (unpaired) for groups of two and one-way ANOVA (followed by Newman–Keuls multiple comparison test) for groups of three or more.

## Results

### Increased Plasma Levels of CS in Diabetic Mouse Models and Patients

**FIGURE 1:**
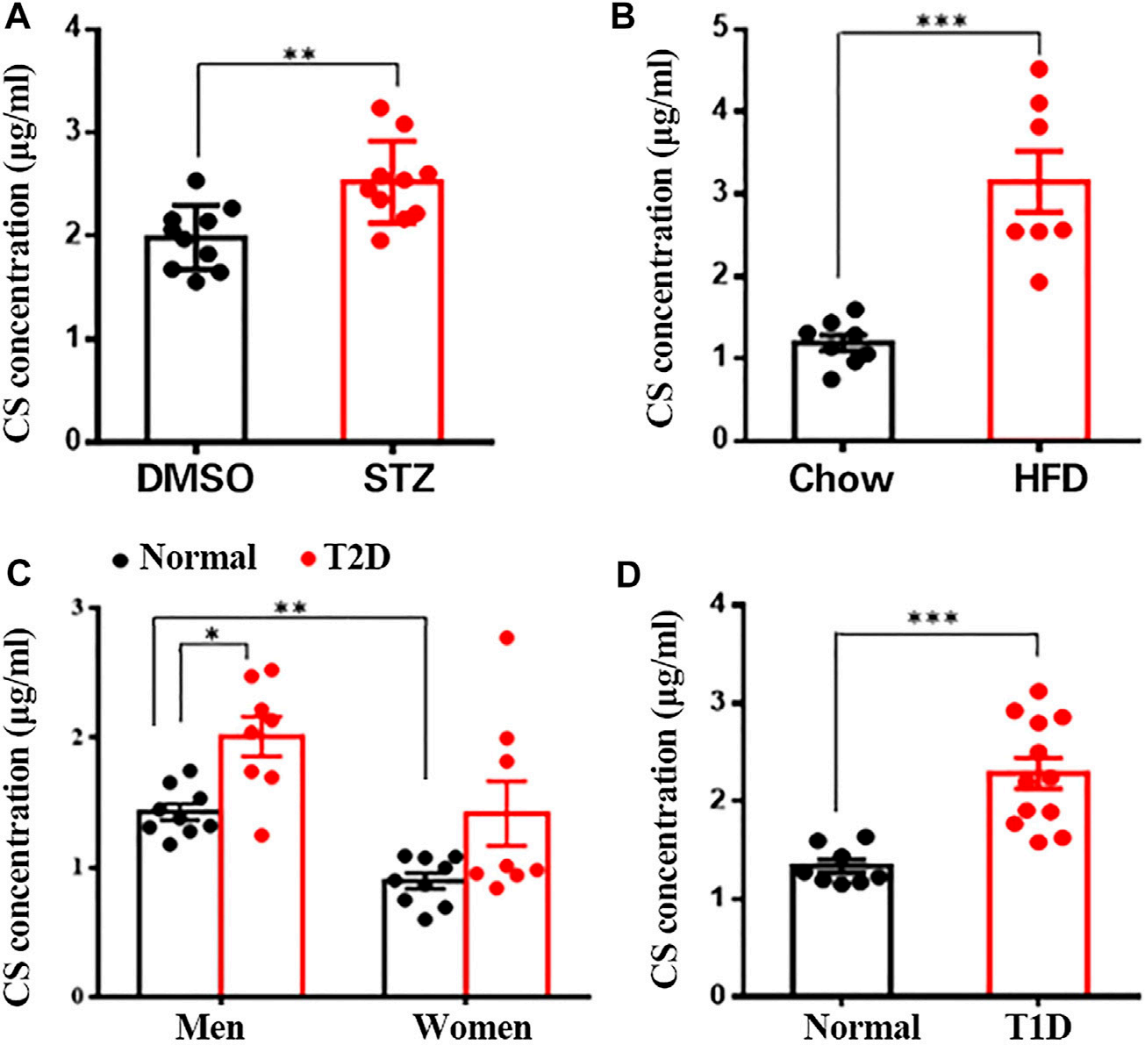
CS levels were increased in blood of diabetic mouse models and patients. Plasma CS levels were determined using a LC-MS/MS. **(A)** Eight-week-old male mice were subcutaneously injected with DMSO daily for 42 days (control group, *n* = 10), mice were injected subcutaneously STZ (40 mg/kg of body weight daily) for four continuous days from the 8th day of the experimental treatment period (STZ group, *n* = 8). **(B)** Eight-week-old male mice were fed with either a normal chow diet or a high-fat diet (HFD) for 8 weeks. **(C,D)** Blood samples from type 2 diabetic (T2D) patients (eight men and eight women) **(C)**, type 1 diabetic (T1D) patients (12 men) **(D)** and age-matched healthy volunteers were collected after an overnight fast. *p < 0.05, **p < 0.01, ***p < 0.001.

Since CS is a major sterol sulfate in plasma, we wanted to determine the potential changes in plasma CS content under various pathologic conditions linked to diabetes. Clinically, multiple low doses of STZ induce a mild impairment of insulin secretion that is more similar to the later stages of T2DM (Srinivasan et al., 2005). Compared to the control mice, STZ-treated mice showed markedly increased fasting blood glucose (Supplementary Figure S1B) and diminished serum insulin concentration (Supplementary Figure S1C), paralleled by significantly lower body weight (Supplementary Figure S1D). Additionally, other characteristic diabetic symptoms, such as polyphagia and polydipsia, were also observed in these mice (Supplementary Figures S1E,F). With respect to plasma CS level, this parameter was significantly higher in the STZ group than in the control group (2.52 ± 0.13 μg/ml vs. 1.98 ± 0.10 μg/ml, *p* < 0.01) (Figure 1A). The HFD-induced obese mouse is generally accepted as a T2DM model (Heydemann 2016) displaying hyperglycemia and a significantly higher body weight (Supplementary Figures S1G,H), and the plasma CS level was detected to be remarkably higher than that in the lean mice (3.15 ± 0.37 μg/ml vs. 1.19 ± 0.10 μg/ml, *p* < 0.001) (Figure 1B).

Subsequently, we wanted to know whether the level of CS is changed in diabetic patients. As expected, fasting blood glucose level was significantly higher in both type 1 and 2 diabetic patients than in the corresponding sex- and age-matched normoglycemic controls (Supplementary Figures S1I,J). Quantitative analysis of plasma CS revealed a significantly higher CS level in men than in women (1.44 ± 0.07 μg/ml vs. 0.89 ± 0.06 μg/ml, *p* < 0.01), and male T2D patients exhibited a markedly elevated CS level than control subjects (1.99 ± 0.17 μg/ml vs. 1.44 ± 0.07 μg/ml, *p* < 0.01) (Figure 1C). Although there was no significant difference in women (1.38 ± 0.31 μg/ml vs. 0.89 ± 0.06 μg/ml, *p* > 0.05), the plasma CS level did show an increasing tendency in diabetic patients (Figure 1C). Furthermore, the plasma CS level was found to be markedly increased in T1D patients compared to the controls (2.29 ± 0.16 μg/ml vs. 1.34 ± 0.07 μg/ml, *p* < 0.001) (Figure 1D). Collectively, these data suggest a correlation between increased plasma CS level and hyperglycemia.

### CS Reduces Diabetic Hyperglycemia in STZ-Induced Diabetic Mouse Models

**FIGURE 2:**
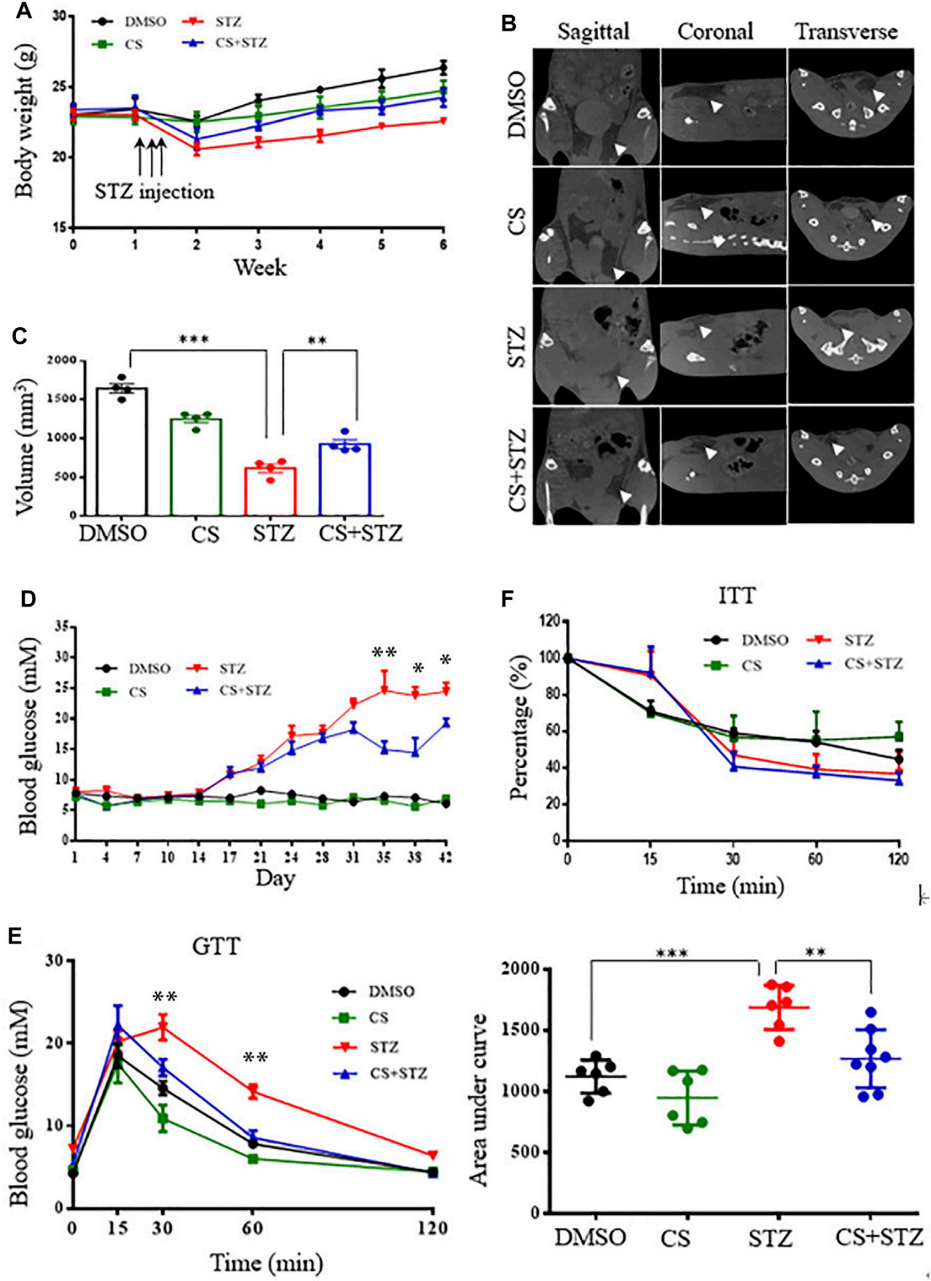
CS relieves diabetes in streptozotocin (STZ)-treated mice. Eight-week-old mice were subcutaneously injected with DMSO or CS (25 mg/kg/dose, two doses daily) for 42 continuous days. Mice were administrated subcutaneously with STZ (40 mg/kg of body weight daily) for four continuous days from the 8th day of the experimental treatment period. **(A)** Body wight was monitored over time (*n* = 7–10 mice per group). **(B,C)** Body fat was measured by micro-computed tomography **(B)** and adipose tissue volumes between lumbar vertebra one and the caudal vertebra four were compared **(C)** at the end of the experiment. **(D)** Blood glucose levels were determined using a glucometer from the tail vein every two other days. **(E)** Glucose tolerance tests (GTT) were performed in 12-h fasted mice given intraperitoneally glucose (2 g/kg body weight). Plasma glucose concentrations were measured at the designated time points (left chart) and the areas under the curve (AUC) of GTT were calculated (right chart) (*n* = 6–8 for each group). **(F)** Insulin tolerance tests (ITT) were conducted in 6-h fasted mice with an intraperitoneal injection of insulin (1 unit/kg body weight). The percentages are relative variations of plasma glucose concentrations at the designated time points from the time 0 min, which was arbitrarily set as 100% (*n* = 8–10 mice per group). *p < 0.05, **p < 0.01, ***p < 0.001.

To determine whether CS mediates glucose homeostasis, we examined its effects in mice treated with multiple low-dose STZ-induced diabetes. From the second week onwards, the body weight of mice in the STZ and CS + STZ groups was significantly lower than that of the control group (Figure 2A). However, the mice treated with CS had a significantly increased body weight compared to that of the mice in the STZ group (Figure 2A). Further evidence demonstrated that CS can relieve STZ-induced adipose tissue loss in mice at the end of the experiment (Figures 2B,C, Supplementary Figure S2). The blood glucose levels of mice in the CS + STZ group and CS group were not different during the first 30 days, but they were significantly higher than those of control mice. On the 31st day, the mice in the CS + STZ group exhibited significantly lower fasting blood glucose levels than those in the STZ group (Figure 2D). The GTT results showed that control mice were glucose tolerant, whereas STZ mice had an impaired tolerance to glucose (Figure 2E), the area under the blood glucose curve (AUC) was increased by 54.5 ± 1.8% in the STZ group compared to that in the control group (Figure 2E). CS-treated mice exhibited a remarkably improved glucose tolerance, and the AUC in the GTT was reduced by approximately 29.2 ± 2.3% in the STZ + CS group compared with that in the STZ group (Figure 2E). However, the effectiveness of insulin in lowering blood glucose, as shown in the ITT, was comparable between mice treated with STZ + CS and STZ alone (Figure 2F). Together, these results indicate that CS could alleviate the STZ-induced impairment of glucose homeostasis but had no effect on systemic insulin sensitivity.

### CS Promotes β-Cell Survival Under Stress

**FIGURE 3:**
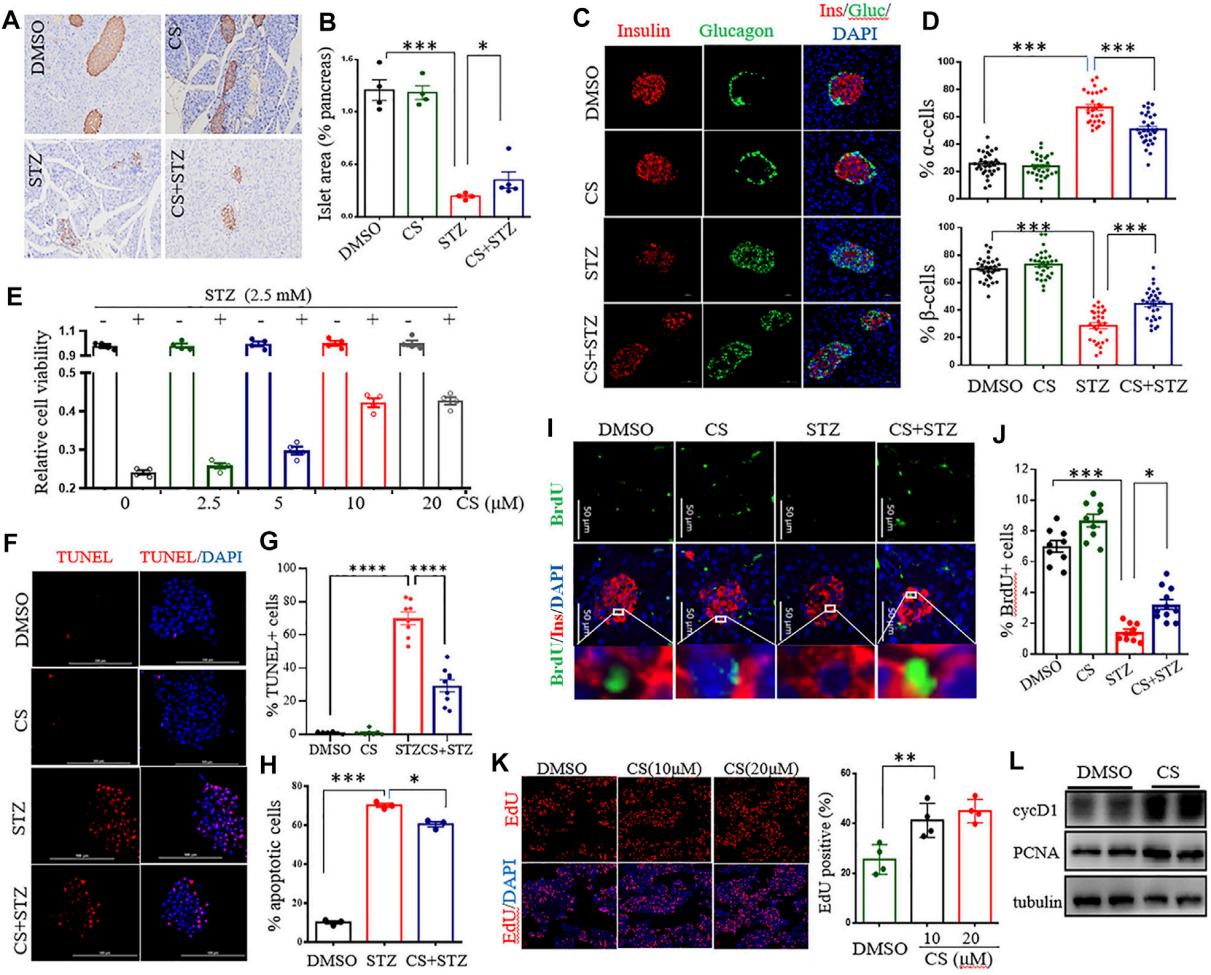
CS preserved β-cell viability by enhancing cell proliferation and suppressing apoptosis. **(A–D)** Eight-week-old mice were subcutaneously injected with DMSO or CS (25 mg/kg body weight/dose, two doses daily) for 42 continuous days. STZ (40 mg/kg of body weight/day) was administrated subcutaneously daily for 4 days from the eighth day of the experimental treatment period. **(A)** Example immunohistochemical staining for insulin on formalin-fixed paraffin-embedded pancreas, insulin staining appears brown. **(B)** Quantitative assessment of the islet area relative to the total pancreas. Each dot represents an individual mouse, one random pancreas section per mouse was examined (*n* = 4–5 mice per group). **(C)** Maintained islet architecture as assessed by immunostaining analysis of pancreatic cryosections with antibodies against insulin (β cells, red), glucagon (α cells, green) and DAPI (nuclei, blue). Scale bars = 50 μm. **(D)** Quantitative comparison of the proportion of glucagon-positive (upper chart) and insulin-positive (lower chart) cells in mice pancreatic islets. 25–40 islets were analyzed for each group (*n* = 4 mice per group). **(E)** Effects of CS on viability of INS-1 β-cells exposed to STZ. Following preincubation with the indicated concentration of CS for 6 h, INS-1 cells were coincubated with CS and 2.5 mM of STZ for 12 h. Cell viability was determined by CCK-8 assay. **(F–H)** Effects of CS on β-cell apoptosis. **(F,G)** Isolated mouse islets were treated with DMSO or CS (10 µM) or STZ (2.5 mM) or a coincubation of CS and STZ for 12 h β-cell apoptosis was measured by TUNEL and represented by percentage of TUNEL positive for total cells. **(H)** INS-1 β-cell apoptosis was detected using a flow cytometric assay with Annexin V and PI staining. **(I–K)** Effects of CS on β-cell proliferation. **(I,J)** Double staining for BrdU (green) and insulin (red) of pancreatic β-cells from mice treated with CS and/or STZ as indicated in panels **(A–D, I)**, and the proliferation of β-cells was quantified as the number of nuclei from both BrdU- and insulin-positive cells divided by the number of nuclei from only insulin-positive cells **(J)**. **(K)** The proliferation of INS-1 β-cells was measure by EdU incorporation staining from four to six independent experiments. **(L)** The expression of cell cycle regulators was determined by Western blotting. *p < 0.05, **p < 0.01, ***p < 0.001.

CS reduced STZ-induced diabetic hyperglycemia without an alternation of the insulin sensitivity, indicating preservation of islet β-cell mass and function in these mice. We next examined the morphology of the pancreatic islets. STZ-treated mice exhibited a markedly decreased insulin-positive area and a severe reduction in the β-cell proportion in the islets, with an expansion of α-cells infiltrating into the center of the islets (Figures 3A–D). Daily CS injections initiated 7 days before STZ treatment partially prevented β-cell loss, resulting in a preservation of the β-cell mass and a reduction of the α-cell infiltration by 15.9 ± 2.2% (Figures 3C,D). However, the α-cell arrangement did not recover to the normal peripheral positioning pattern (Figure 3C).

The protection of CS against STZ-induced β-cell loss could arise from a change in β-cell viability or proliferation. To determine whether CS influences β-cell survival under stressful conditions, cell viability was investigated in STZ-exposed INS-1 β-cells. STZ at a concentration of 2.5 mM markedly decreased INS-1 cell viability by 51.4 ± 6.5% (*p* < 0.001) (Supplementary Figure S3A). However, a preincubation with CS alleviated STZ-induced cell death in a dose-dependent manner (Figure 3E), and 10 μM CS significantly ameliorated STZ-induced cell death by 22 ± 5.9% (*p* < 0.05). In addition, CS exposure also protected β-cells against oxidation (H_2_O_2_)_-_ or lipitoxity (palmitic acid, PA)- or glucotoxity (high concentration glucose, HG)-induced cell death at concentrations of 2.5 μM or 5 μM, respectively (Supplementary Figures S3B–F). These results thus unequivocally demonstrated that CS improves β-cell viability under stress.

Subsequently, we determined whether CS could protect β-cells against apoptosis in primary islets and INS-1 β-cells. STZ significantly induced apoptosis tending to a 67.3 ± 5.4% increase in primary islets (Figures 3F,G). This proapoptotic effect was significantly suppressed by CS, and 10 μM CS inhibited apoptosis by 32.3 ± 7.2% (*p* < 0.01) (Figures 3F,G). Furthermore, CS exposure at a concentration of 10 μM also significantly attenuated STZ- or H_2_O_2_- or PA- or HG-induced apoptosis by 8.5 ± 1.4% (*p* < 0.01), 11.8 ± 1.2% (*p* < 0.01), 7.7 ± 1.4% and 9.6 ± 1.3% (*p* < 0.01) of INS-1 β-cells, respectively (Figure 3H, Supplementary Figures S3G–I). Moreover, we determined the impact of CS on pancreatic β-cell proliferation as defined by BrdU incorporation into insulin positive cells. As shown in Figures 3I,J, STZ-treated mice showed a markedly reduced β-cell proliferation (*p* < 0.001), daily CS injections initiated 7 days before STZ treatment led to a significant increase of β-cell proliferation (*p* < 0.05). We further performed an EdU incorporation assay in the INS-1 cell line. We found that 10 μM CS significantly increased the number of EdU^+^ cells (Figure 3K). Simultaneously, the protein levels of PCNA and cyclin D1 implicated in β-cell proliferation were significantly upregulated in these cells (Figure 3L). Collectively, these observations suggest that CS promotes both β-cell replication and survival.

### CS Promotes β Cell Survival by Activating CREB

**FIGURE 4:**
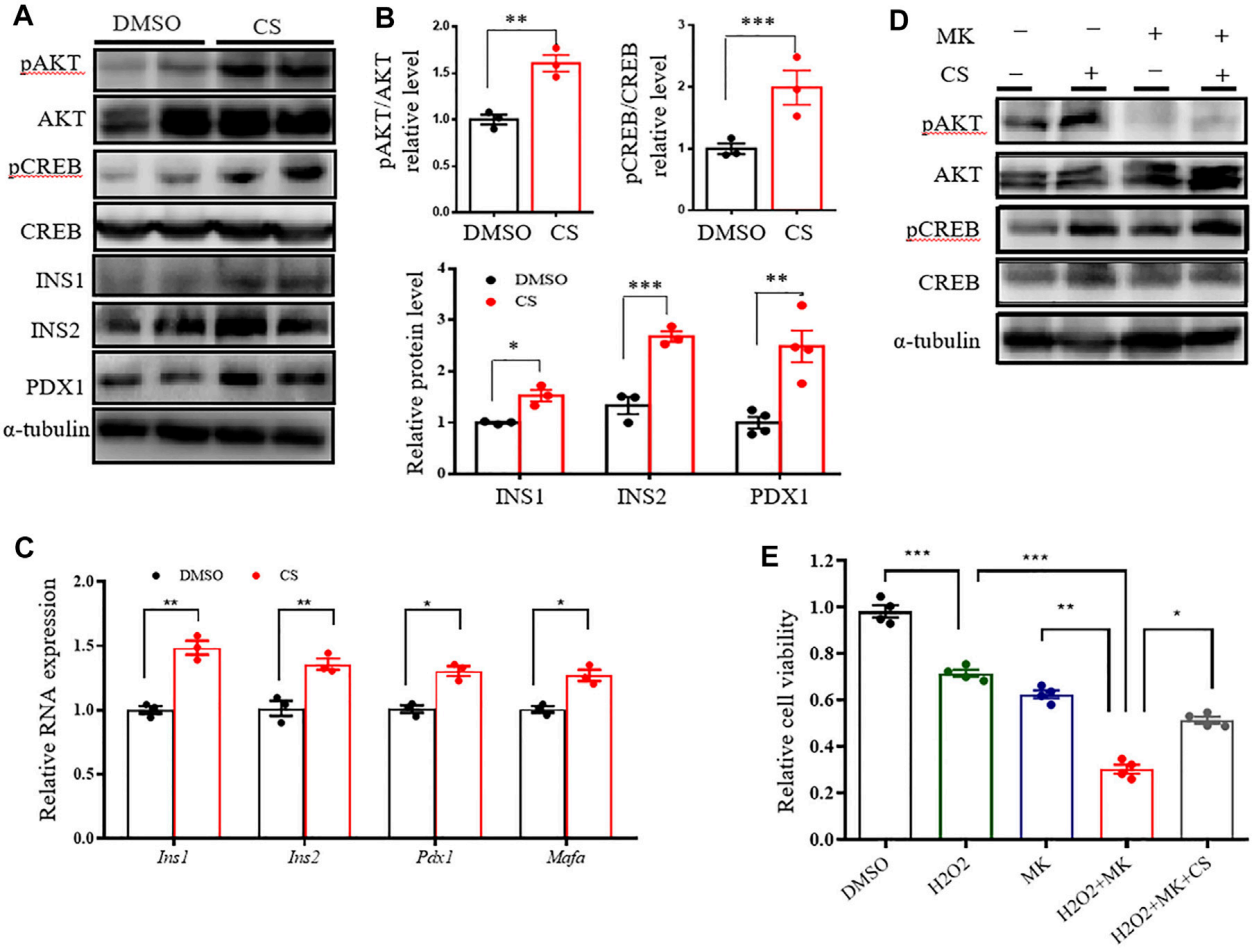
CS activated CREB independently of AKT in INS-1 β-cells. **(A–C)** INS-1 cells were stimulated with DMSO or CS (10 μM) for 12 h. Protein levels of pAKT, AKT, pCREB, CREB, INS1, INS2 and PDX1 were measured by Western blotting **(A,B)** and mRNA expressions of *Ins1*, *Ins2*, *Pdx1* and *Mafa* were determined by quantitative PCR **(C)**. **(D)** INS-1 cells were treated with CS (10 μM) for 12 h in the presence or absence of AKT inhibitor MK2206 (5 μM), the latter was added 30 min prior to CS treatment. Protein levels of AKT and CREB and their phosphorylation were determined by Western blotting. **(E)** INS-1 cells were incubated with 10 μM CS for 1 h in the presence or absence of 5 μM MK2206 (AKT inhibitor). Thereafter, cells were coincubated with CS and 2.5 mM STZ for 24 h. Cell viability was determined by CCK-8 assay. Experiments were done in quadruplicate in each treatment. *p < 0.05, **p < 0.01, ***p < 0.001.

Since the AKT signaling pathway is critical in the regulation of human and rodent pancreatic β-cell survival and islet mass (Yuan et al., 2018; Tuttle et al., 2001), we tested the impact of CS on the activation of the AKT pathway. Western blotting showed that CS markedly promoted AKT activation in INS-1 β-cells (Figures 4A,B). cAMP-response element–binding protein (CREB), a crucial transcription factor for β-cell gene expression and function (Dalle et al., 2011; Blanchet et al., 2015), is one of the crucial downstream effectors of AKT (Manning and Toker 2017). We next determined the levels of CREB protein and its serine 133 phosphorylation, which is required for CREB-mediated transcription. We found that CREB was remarkably phosphorylated in CS-treated cells (Figures 4A,B). In particular, this was simultaneously associated with an increased abundance of proteins and transcripts of insulin (Ins1 and Ins2), along with β-cell markers of Pdx1 and Mafa (Figures 4A–C). However, our data also showed that pharmacological blockade of Akt activation did not attenuate CS-induced CREB phosphorylation (Figure 4D, Supplementary Figure S4A). Consistent with these observations, CS protected INS-1 cells against H_2_O_2_- or PA-induced cell death even when the cells were exposure to the AKT blockade (Figure 4E, Supplementary Figure S4B). These results suggested that CS stimulates β-cell signaling through a mechanism involving the independent activation of the AKT and CREB pathways.

### CS Restores Insulin Secretion From β Cells

**FIGURE 5:**
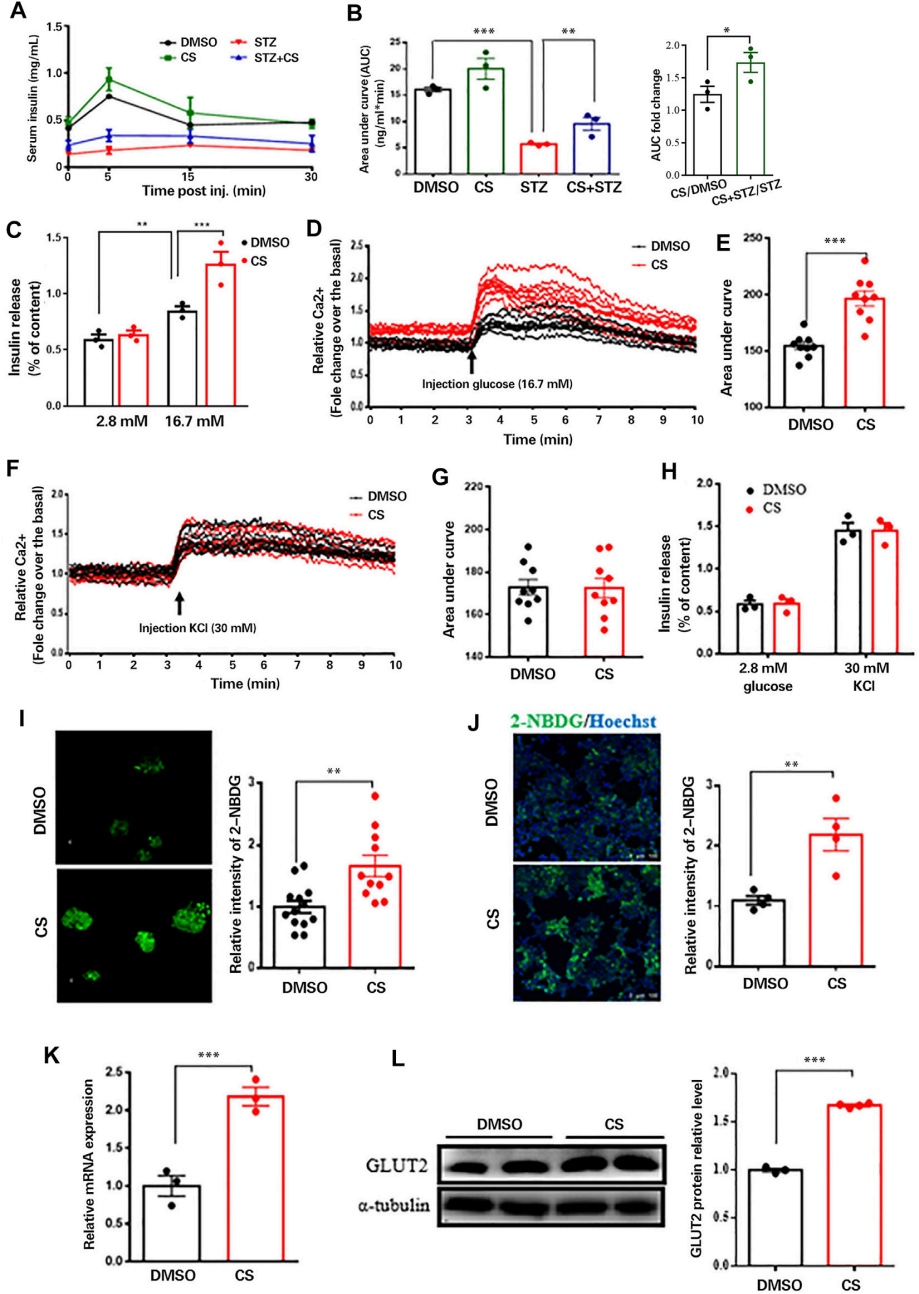
CS restored insulin secretion from β-cells response to glucose not to depolarizing secretagogues. **(A)** Plasma insulin concentrations were measured at indicated time points during glucose-stimulated insulin secretion (GSIS) in response to administration of 3 g glucose/kg body weight in 12-h fasted mice (*n* = 4–6 mice per group). **(B)** AUC calculated from GSIS data were compared. **(C)** Insulin secretion measured in isolated islets challenged with 2.8 or 16.7 mmol/L glucose. **(D–G)** Mouse islets were pretreated with or without CS (10 µM) for 12 h followed by a perfusion with KRB buffer-based solutions containing 16.7 mM glucose **(D,E)** or 30 mM KCl **(F,G)**, intracellular Ca^2+^ were determined by ratiometric Fura-3AM fluorescence measurements. Change of intercellular Ca^2+^ contents **(D,F)** and the relative islet Ca^2+^ influx (AUC) during 10-min stimulation **(E,G)**. **(H)** Insulin release from isolated islets pretreated with or without CS (10 µM) for 12 h followed by a challenge of 30 mM KCl in 2.8 mM glucose buffer. **(I,J)** Glucose uptake ability in isolated islets **(I)** or INS-1 β-cells **(J)** was measured using 2-NBDG. Mouse islets or INS-1 β-cells were incubated with DMSO or 10 µM CS for 12 h followed by a 30-min incubation of 100 μg/ml 2-NBDG in KRB buffer, fluorescent images were measured with an excitation wavelength of 475 nm and an emission wavelength of 550 nm. **(K,L)** Effects of CS on *Glut2* mRNA expression **(K)** and GLUT2 protein levels **(L)** were determined by quantitative PCR and Western blotting, respectively. *p < 0.05, **p < 0.01, ***p < 0.001.

We next wished to delineate whether the protective effect of CS on STZ-induced pancreatic β-cell injury is concomitant with β-cell functional restoration. To this end, we first performed a glucose-stimulated insulin secretion (GSIS) assay *in vivo*. As shown, CS partially but significantly restored the STZ-induced declination of insulin secretion in response to glucose stimulation (Figure 5A), and the area under the insulin secretion curve (AUC) was increased by 65.2 ± 4.6% in CS-exposed mice compared to that in STZ-treated mice (Figure 5B). We next examined insulin secretion from isolated islets. Despite a comparable basal insulin release between CS-exposed islets and DMSO-treated control islets, the insulin secretion was significantly elevated in islets exposed to CS in comparison with the control islets after being challenged with high-concentration glucose (16.7 mM) (Figure 5C). Together, these results suggest that CS preserves the ability of GSIS in STZ-injured islet β-cells.

Since glucose stimulates insulin secretion by inducing depolarization and Ca^2+^ influx (Yang et al., 2020), we measured intracellular Ca^2+^ levels in perfused islets exposed to 16.7 mM glucose. Glucose-evoked Ca^2+^ influx was significantly increased in islets with CS exposure compared with control islets (Figures 5D,E). β-Cell Ca^2+^ entry is induced by the closure of ATP-sensitive potassium channels (K_ATP_ channels) with consequent depolarization; therefore, we examined the potential effect of CS exposure on the activity of K_ATP_ channels in β-cells. Ca^2+^ influx induced by 30 mM KCl in the presence of basal 2.8 mM glucose was equivalent between CS-exposed and control islets (Figures 5F,G). Consistent with these observations, insulin secretion was comparable between CS-treated and control islets challenged with KCl (Figure 5H). These findings indicated that the enhanced GSIS in CS-exposed islets was independent of K_ATP_ channels, and signaling events upstream of membrane depolarization appear to be responsible for CS-modulated insulin secretion.

In an attempt to identify the upstream event by which CS regulates GSIS, we evaluated whether CS can increase glucose uptake into β-cells, which was monitored by the uptake of 2-NBDG. As shown, glucose uptake was significantly elevated in CS-exposed islets compared to DMSO-treated control islets (Figure 5I). This was further confirmed in INS-1 β-cells exposed to CS (Figure 5J). These findings strongly suggested that CS-enhanced Ca^2+^ entry and GSIS result from increased glucose uptake. Given that blood glucose is taken up by β-cells mainly through GLUT2 (Thorens 2015), we postulated that CS might increase GLUT2. Indeed, both the mRNA expression (Figure 5K) and protein levels (Figure 5L) of GLUT2 were markedly upregulated in cells exposed to CS. Thus, we concluded that CS-induced insulin secretion was at least partially mediated by increases in GLUT2 levels and GLUT2-mediated glucose uptake.

### CS Preserves Mitochondrial Function in Islet β-Cells Under Stress

**FIGURE 6:**
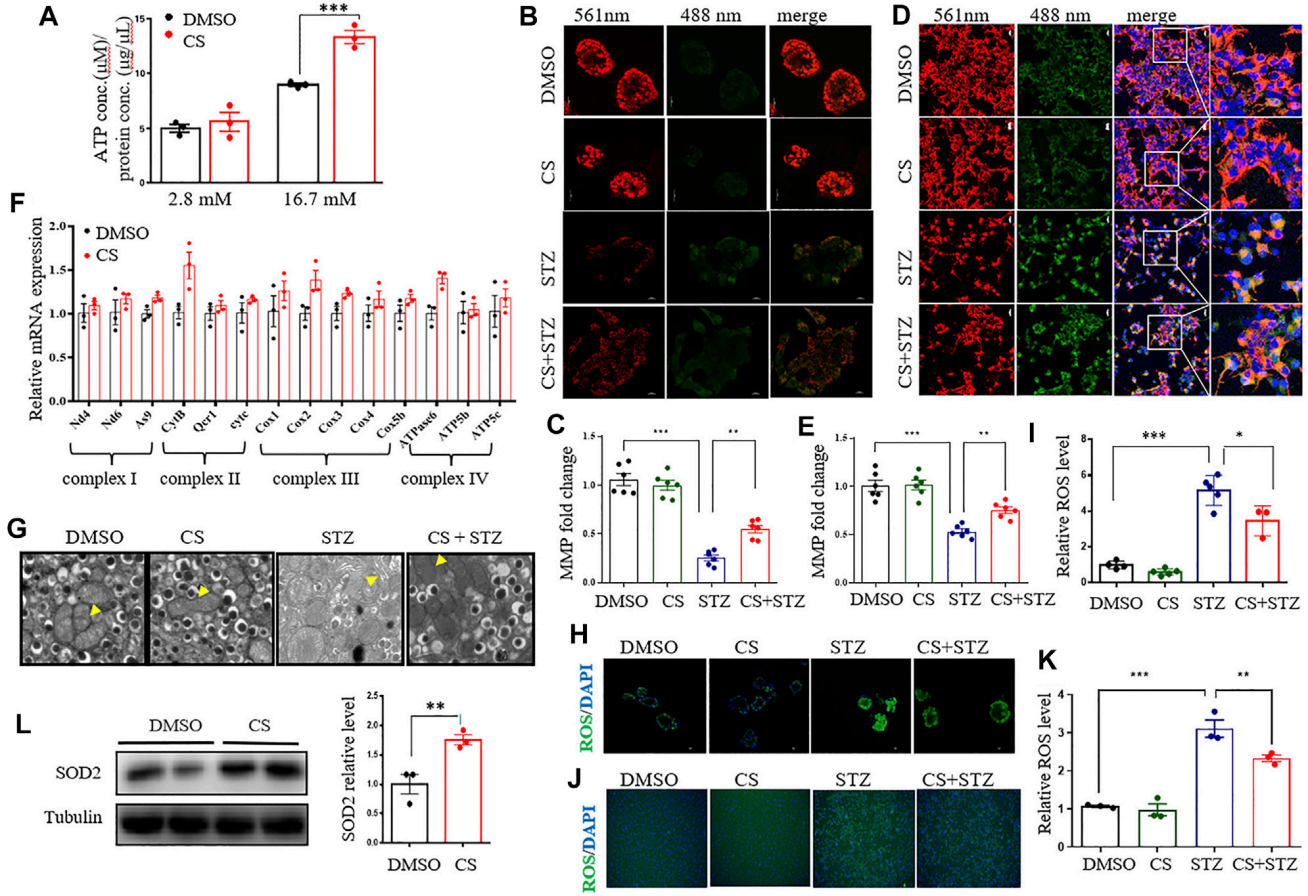
CS restores mitochondrial function of islets under stressful condition. **(A)** Total cellular ATP concentration in isolated islets was determined following a 20-min incubation with glucose at the concentration of 2.8 mM or 16.7 mM. **(B–E)** Mitochondrial membrane potential (MMP) was assayed in mouse islets **(B,C)** or INS-1 β-cells **(D,E)** after a 6-h preincubation of 10 µM CS or DMSO followed by a coincubation of CS (10 µM) and STZ (2.5 mM) for 12 h. JC-1 sends red or green fluorescence when MMP is high in the normal mitochondria or low in the unhealthy mitochondria, respectively. Representative images were taken at emission 488 nm (green-indicating JC-1 monomers in the cytoplasm) and at 561 nm (red-indicating JC-1 polymers in the mitochondrial matrix). **(F)** Relative mRNA expressions of genes encoding mitochondrial respiration complex factors in INS-1 β-cells treated with DMSO or 10 µM CS for 12 h. Individual measurement was normalized to 18s rRNA expression, and the DMSO-treated control average was set to 1. **(G)** Mouse islets sections were examined by transmission electron microscope (TEM) (scale bar: 1 µm). The yellow triangles indicate swollen mitochondria around the nucleus. **(H–K)** Effects of CS on STZ-induced reactive oxygen species (ROS) in mouse islets **(H,I)** and INS-1 β-cells **(J,K).** Mouse islets or INS-1 β-cells were incubated with 10 µM CS for 6 h followed by a 12-h incubation with 2.5 mM STZ. ROS was measured with a ROS assay kit. The fluorescence signal was monitored with a 448-nm excitation filter and fluorescence intensity was quantified. **(L)** The protein levels of SOD2 were determined by Western blot. *p < 0.05, **p < 0.01, ***p < 0.001.

Increased oxidative metabolism of glucose in the mitochondria and the resultant elevated cellular ATP synthesis are key steps in the regulation of glucose-induced insulin release in β-cells (Las et al., 2020; Liu et al., 2021). Although the intracellular ATP content was significantly increased in both CS- and DMSO- treated islets under high glucose (16.7 mM) conditions compared to low glucose (2.8 mM) conditions, CS exposure markedly increased ATP content relative to DMSO treatment (Figure 6A). Given that mitochondrial ATP synthesis in pancreatic β-cells is driven by a change in mitochondrial membrane potential (MMP) in response to glucose stimulation, we then detected the effect of CS on the MMP of β-cells by using JC-1. The degree of depolarization of mitochondria can be measured by the ratio of red/green fluorescence intensity. As shown, CS markedly attenuated the STZ-induced reduction in MMP in both isolated mouse islets (Figures 6B,C) and INS-1 β-cells (Figures 6D,E), indicating that CS can preserve MMP under stressful conditions. This was further confirmed in islets or INS-1 β-cells challenged with either H_2_O_2_ or PA (Supplementary Figure S5). Additionally, the expression of mRNAs encoding mitochondrial respiratory complex components, such as As9, CytB, Cox2 and atpase6, showed an increased trendency in CS-exposed islets relative to control islets (Figure 6F), suggesting an improved mitochondrial function in β-cells after CS exposure. Further ultrastructural analysis by TEM revealed that STZ-exposed β cells exhibited serious mitochondrial structural abnormalities characterized by marked intracristal swelling and reduced electron density, which were significantly alleviated by CS exposure (Figure 6G). Overall, these data indicate that CS can maitain mitochondrial integrity in islet β-cells.

To further gain insight into the possible mechanisms underlying the effect of CS on mitochondrial homeostasis, we examined its effect on mitochondrial fission and fusion, which are important processes for functional mitochondria after cell damage (Dorn and Kitsis 2015). In INS-1 β-cells, exposure to STZ or PA stimulated a partial mitochondrial fission process as evidenced by the alternation of mitochondrial shape from wirelike to shorter and granular, and CS could not prevent this process (Supplementary Figure S6). As mitochondrial ROS generation is a hallmark of STZ- or PA-induced islet toxicity, we also determined whether CS impacts ROS release. In isolated mouse islets, both STZ and H_2_O_2_ markedly increased ROS release (*p* < 0.001), while CS showed a significantly effective anti-ROS effect (Figures 6H,I, Supplementary Figure S7A). This was further confirmed in INS-1 β-cells. When INS-1 cells were exposed to STZ or H_2_O_2_, mitochondrial ROS increased and was significantly suppressed by CS (Figures 6J,K, Supplementary Figure S7B). Furthermore, the expression of the antioxidant protein SOD2 was significantly increased in CS-treated INS-1 cells (Figure 6L).

## Discussion

**FIGURE 7:**
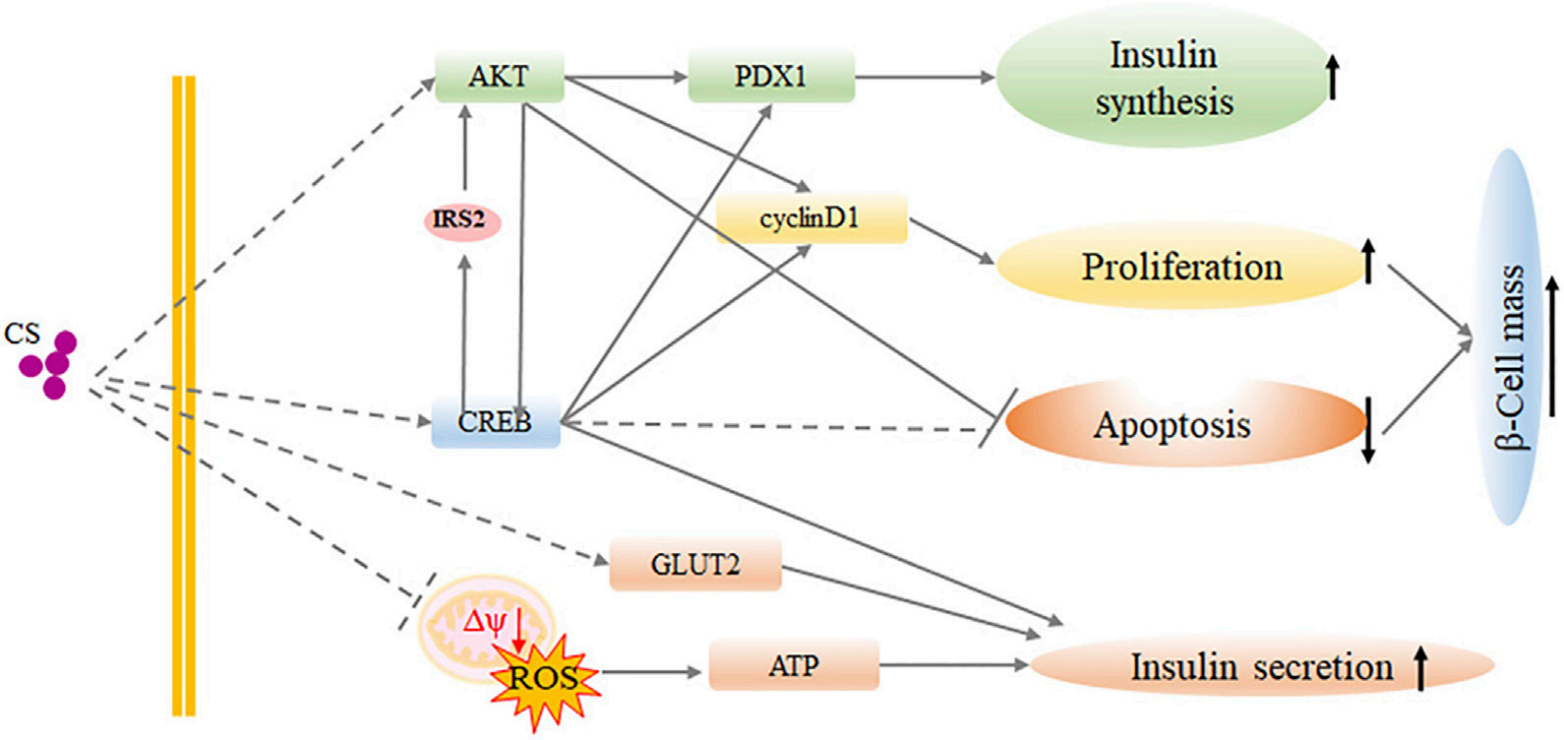
Schematic representation of CS function in β-cell proliferation and function.

CS has emerged as a significant lipid constituent in a variety of human tissues, and it acts as a key player of in many biological pathways influencing human health and disease (Sanchez et al., 2021). In the present study, we provided evidence that CS contributes to glucose homeostasis by maintaining β-cell mass together with improving β-cell function. It is demonstrated that plasma CS was significantly elevated in diabetic conditions. CS therapy partially alleviated diabetes in a multiple low-dose STZ-induced diabetic mouse model, which is similar to the later stage of type 2 diabetes with a mild impairment of insulin secretion. Thus, CS might be beneficial at a very early stage of the disease where the β-cell mass is still sufficient and the threshold for reversibility of decreased β-cell mass has not been exceeded. Mechanistically, CS increased β-cell proliferation and protected β-cells against apoptosis, which in turn increased β-cell mass. Importantly, CS supported GLUT2 expression and mitochondrial integrity, which then resulted in an increase in ATP production, thereby enabling insulin secretion machinery in the islets to function adequently (Figure 7). This is the first study revealing a novel dual role of CS in integrating β-cell survival and cell function, which appear to be physiologically relevant and could be applicable to the processes of T2DM.

CS has an important role in the substrate specificity of phosphoinositide 3-kinase (PI3K) (Woscholski et al., 1995). AKT is generally considered as the main target of PI3K, and it is known that the PI3K-AKT signaling pathway is of importance in the regulation of β-cell mass and function (Gu et al., 2011). Previous studies have shown that CS attenuates hippocampal cell apoptosis by activating the PI3K/AKT signaling pathway (Prah et al., 2019). In our study, we observed that CS significantly increased the activation of AKT in pancreatic β-cells. Our observations also showed that CS stimulated the activation of CREB, which is a key transcription factor in the maintenance of efficient glucose sensing, β-cell survival, insulin gene transcription and insulin exocytosis (Dalle et al., 2011; Blanchet et al., 2015). Activation of CREB, in response to a diverse of stimuli including glucose or incretin hormone glucagon-like peptide 1 (GLP-1), stimulates the transcription of β-cell genes that promote islet viability and insulin secretion (Van de Velde et al., 2011). Depletion of CREB activity in pancreatic β-cells leads to hyperglycemia due in part to diminished expression of IRS-2, excessive β-cell apoptosis and decreases in insulin secretion (Withers et al., 1998; Jhala et al., 2003; Blanchet et al., 2015). Previous findings have revealed that CREB is one of the key downstream effectors of the AKT signaling pathway (Manning and Toker 2017). Unexpectedly, our study showed that the CS-induced activation of CREB was not suppressed by the inhibition of the AKT signaling pathway, suggesting that both signaling pathways are actively involved in conveying CS actions in β-cells.

Pancreatic β-cells are susceptible to injury under glucolipotoxicity or inflammatory cytokine-producing conditions. This appears to be due at least in part to the production of ROS causing β-cell apoptosis and a loss of functional β-cell mass (Vela-Guajardo et al., 2021). We found that CS contributed to the rescue of STZ- or H_2_O_2_-impaired β-cell mitochondrial respiration and ATP generation. This underlines and corroborates previous studies (Han et al., 2014), demonstrating that CS can play a role in the prevention, delaying or treatment of diabetes by making β-cells more resistant to oxidative stress. CS acts as an endogenous ligand of retinoic acid-related orphan receptor a (RORα). Previous findings suggested that RORα has a protective function against oxidative stress by the induction of the antioxidant enzymes such as superoxide dismutase 2 (SOD2) and glutathione peroxidase 1 (GPx1) in rat hepatocytes, the treatment of CS reduced the level of oxidative damage with increased RORα levels (Han et al., 2014). Our study demonstrated that CS also played an essential role in supporting β-cell metabolism and promoting survival under stressful conditions. This protective effect undoubtedly contributes to the increase in β-cell mass and function observed.

Glucose is transported into β-cells through GLUT2, followed by glucose metabolism and the generation of ATP, resulting in the activation of voltage-dependent Ca^2+^ channels and Ca^2+^ influx that in turn triggers the release of insulin granules. Our study demonstrated that CS markedly increased GLUT2 expression and GLUT2-mediated glucose uptake into β-cells. However, evidence also suggests that GSIS can proceed normally even in the presence of low levels of GLUT2 (Thorens et al., 2000; Thorens 2015). Therefore, the stimulatory effect of CS on insulin secretion may not be atributed to the elevation of intracellular GLUT2. MMP, as an essential component of the proton motive force, is essential for ATP production and essential for metabolic coupling factors including reactive oxygen species, which itself is essential for GSIS (Gerencser 2018). We found that CS could alleviate the reduction in MMP in β-cells under stressful conditions. Furthermore, CS increased the expression of genes encoding mitochondrial respiratory chain complex components, indicating a functional improvement of mitochondrial respiration complexes in β-cells. Therefore, it is likely that the improved GSIS in the CS treated STZ-induced diabetic mice is due to the combined increase in GLUT2 and mitochondrial function in β-cells.

Overall, our study provides novel evidence that CS can attenuate diabetes in STZ-induced diabetic mice by increasing both pancreatic β-cell mass and function. It shows that CS modulates mitochondrial efficiency, and by that provides islet cells with enhanced antioxidative responses by which less ROS is generated when β-cells are exposed to stressful conditions. Therefore, it prevents pancreatic β-cell apoptosis and supports cell function. Our findings suggest that the use of CS offers a physiologic approach to preserve the viability of islets and protect against the development of diabetes mellitus.

## Data Availability

The original contributions presented in the study are included in the article/Supplementary Material, further inquiries can be directed to the corresponding author.
