# Supplementary material for: Cholesterol Sulfate Exerts Protective Effect on Pancreatic β-Cells by Regulating β-Cell Mass and Insulin Secretion
Source: Front Pharmacol. 2022 Mar 4;13:840406. doi: 10.3389/fphar.2022.840406 (PMC8930834; doi:10.3389/fphar.2022.840406)
Supplement: Supplementary file 1 [file DataSheet1.docx]

**Cholesterol sulfate exerts protective effect on pancreatic β-cells by regulating β-cell mass and insulin secretion**

Xueping Zhang^1†^, Dan Deng^1†^, Daxin Cui^1†^, Yin Liu^1^, Siyuan He^1^, Hongmei Zhang, Yaorui Xie ^2^, Xiaoqian Yu^1^, Shanshan Yang^1^, Yulong Chen^1^, Zhiguang Su^1^*

1. Molecular Medicine Research Center and National Clinical Research Center for Geriatrics, West China Hospital, and State Key Laboratory of Biotherapy, Sichuan University. Chengdu 610041, China.
2. Department of clinical laboratory, Sichuan Provincial Peoples Hospital Jinniu Hospital. Chengdu 610007, China.

^†^ These authors contributed equally to this work.

*Corresponding author

Zhiguang Su, Ph.D

### Supplementary data consist of Supplementary Figures S1-7 and Tables S1-2.


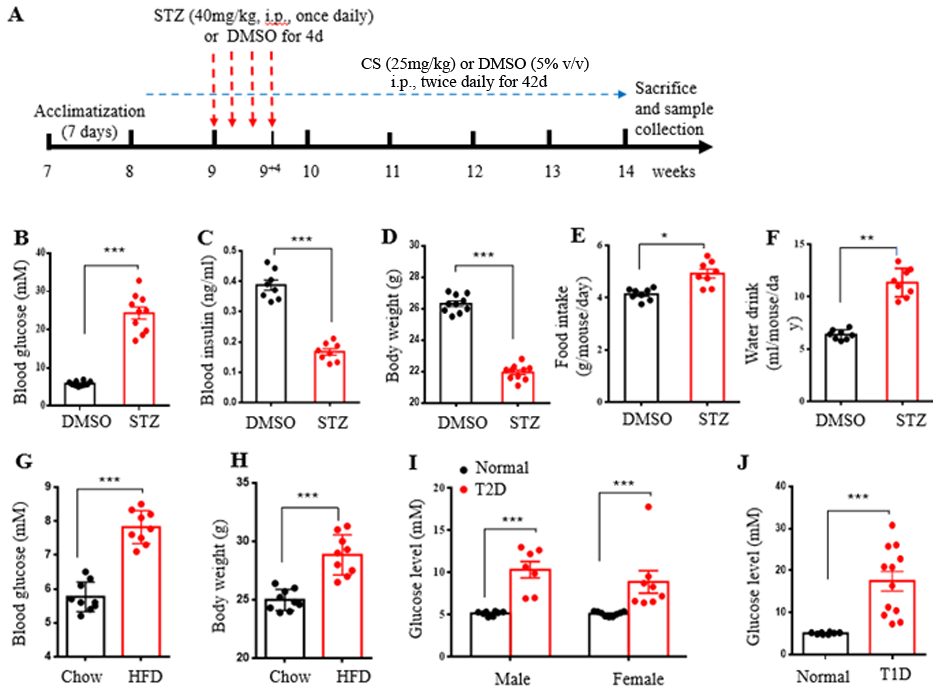


**Figure S1. Characteristic diabetic symptoms in mouse models and patients.** (A) A schematic diagram of the mouse experimental design. After one-week acclimation, 8-week-old male mice were randomly divided into 4 groups (n=10 in each group): the control group (DMSO) with an injection of DMSO (5% v/v), CS-treated group (CS) with a intraperitoneal administration of CS (25 mg/kg body weight/dose, two doses daily), STZ-treated group (STZ) with a 7-day injection of DMSO followed by a 4-day administration of STZ (40 mg/kg of body weight daily, i.p.) and CS + STZ-treated group (CS + STZ ) with a 7-day CS injection followed by a 4-day STZ administration. In the control and STZ-treated groups, DMSO was intraperitoneally injected daily for 42 days, while the CS- and CS+STZ-treated groups were given the same volume of CS twice daily instead of saline. (B-F) Comparisons of metabolic characteristics between mice in DMSO group and STZ-treated group. The levels of blood glucose (B) and serum insulin (C) and body weight (D) were measured at the end of the experiment, food intake (E) and water drink (F) were measured over the experimental treatment period. (G-H) Blood glucose levels (G) and body weight (H) of male mice fed with a high-fat diet (HFD) for 8 weeks at the age of eight weeks old. (I-J) Fasting blood glucose levels of patients with type 2 diabetes (T2D) (I) or type 1 diabetes (T1D) (J).


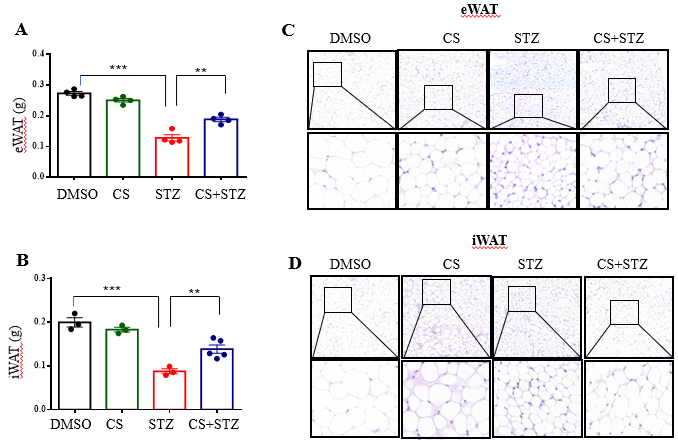


**Figure S2. CS relieves the STZ-induced adipose tissue loss in mice.** Eight-week-old mice were subcutaneously injected with DMSO or CS (25 mg/kg/dose, two doses daily) for 42 continuous days. Mice were administrated subcutaneously with STZ (40 mg/kg of body weight daily) for 4 continuous days from the 8th day of the experimental treatment period. (A-B) Weight of epididymal (A) and inguinal subcutaneous (B) adipose tissue. (C-D) Representative HE-stained sections of epididymal (C) and inguinal subcutaneous (D) adipose tissue.


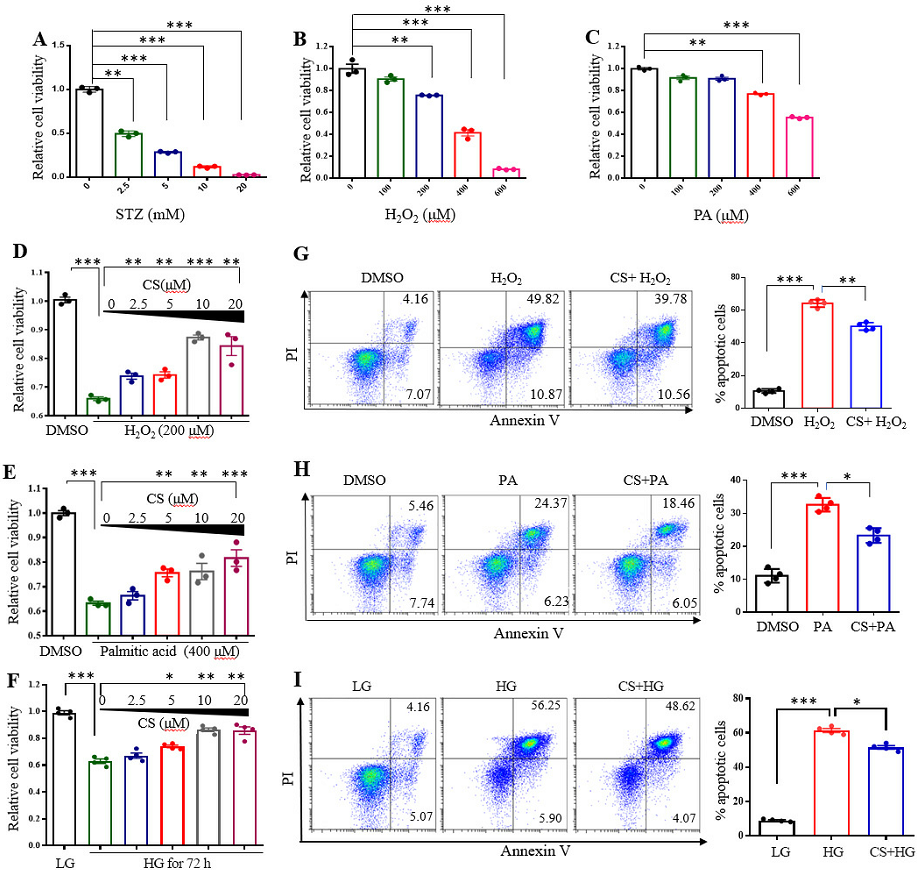


**Figure S3. Effects of CS on viability of INS-1 β-cells under various stressful conditions.** (A-C) INS-1 β-cells were exposed to the indicated concentration of STZ (A), H_2_O_2_ (B) or PA (C) for 12 h. Cell viability was determined by CCK-8 assay. (D-F) Following preincubation with or without the indicated concentration of CS for 6 h, INS-1 cells were coincubated with CS and 200 μM H_2_O_2_ (D) or 400 μM PA (E) for 12 h or high concentration glucose (HG, 33.3 mM) for 72 h (F). Cell viability was determined by CCK-8 assay. (G-I) INS-1 cells were incubated with 10 μM CS for 1 h followed by a 12-h coincubation of CS and 200 µM H_2_O_2_ (G) or 400 µM PA (H) or 33.3 mM glucose (HG) for 72 h (I). Cell apoptosis was detected using a flow cytometric assay with Annexin V and PI staining. Experiments were done in triplicate or quadruplicate in each treatment. **P* < 0.05, ***P* < 0.01, ****P* < 0.001.


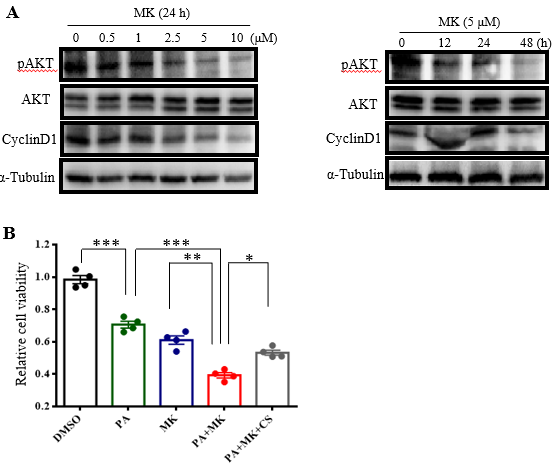


**Figure S4. CS activates AKT and CREB in INS-1 β-cells.** (A) INS1 cells were treated with AKT inhibitor MK2206 at the indicated concentration for 24 h (left) or at the concentration of 5 μM for the indicated time (right). Protein levels of pAKT, AKT and Cyclin D1were measured by Western blotting. (B) INS1 cells were incubated with 10 μM CS for 1 h in the presence or absence of 5 μM MK2206 (AKT inhibitor). Thereafter, cells were coincubated with CS and 400 μM PA for 12 h. Cell viability was determined by CCK-8 assay. Experiments were done in quadruplicate in each treatment. **P* < 0.05, ***P* < 0.01, ****P* < 0.001.


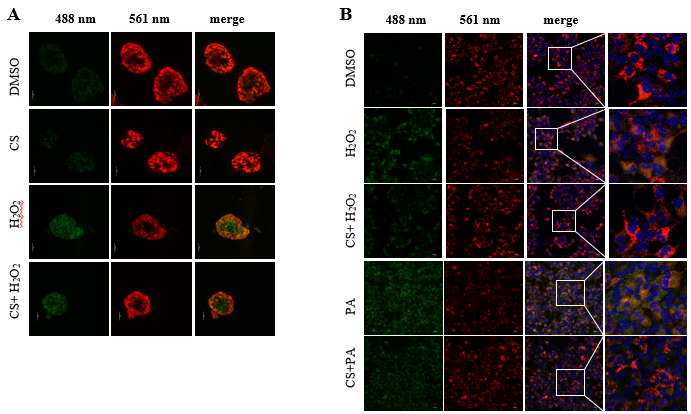


**Figure S5. CS restores mitochondrial membrane potential (MMP) of β-cells** **under stressful conditions.** MMP was assayed using JC-1 fluorescence probe. Mouse islets (A) or INS-1 β-cells (B) were preincubated with CS (10 µM) or DMSO for 6 h, and then were coincubated with 10 µM CS and 200 µM H_2_O_2_ or 400 µM palmitic acid for an additional 12 h. After staining with JC-1, images were taken at emission 488 (green-indicating low MMP in the unhealthy mitochondria) and at 561 nm (red-indicating high MMP in the normal mitochondria).


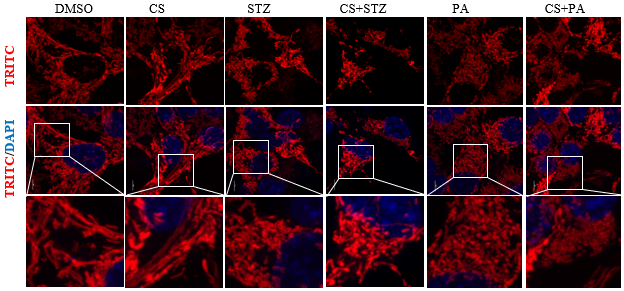


**Figure S6. Effect of CS on mitochondrial shape.** INS-1 β-cells were preincubated with CS (10 µM) or DMSO for 6 h, and then were coincubated with 10 µM CS and 2.5 mM STZ or 400 µM PA for an additional 12 h. Mitochondria were stained with MitoTracker Red (red).


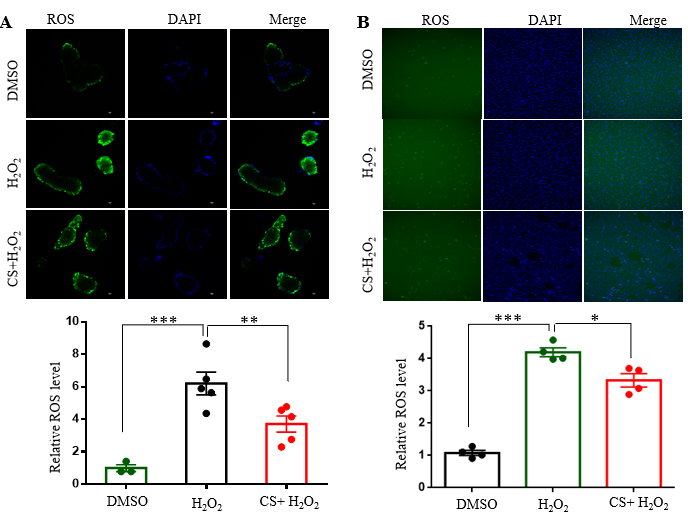


**Figure S7.** **Effects of CS on STZ-induced reactive oxygen species (ROS) in mouse islets and INS-1 β-cells.** Mouse islets (A) and INS-1 β-cells (B) were incubated with 10 µM CS for 6 h followed by a 12-h incubation with 200 mM µM H_2_O_2_. ROS was measured with a ROS assay kit. The fluorescence signal was moINSored with a 448-nm excitation filter (upper) and fluorescence intensity was quantified (lower). **P* < 0.05, ***P* < 0.01, ****P* < 0.001.

**Table S1. Primers used for real-time quantitative PCR analysis**

| Gene | Forward (5' to 3') | Reverse (5' to 3') |
| --- | --- | --- |
| *Cyclin D1* | ATGGAAGGACCCTTGAGGC | CTTCACGGCTTGCTCGTTCT |
| *Cyclin B1* | AAGGTGCCTGTGTGTGAACC | GTCAGCCCCATCATCTGCG |
| *Cyclin D2* | GAGTGGGAACTGGTAGTGTTG | CGCACAGAGCGATGAAGGT |
| *Ins1* | CACTTCCTACCCCTGCTGG | ACCACAAAGATGCTGTTTGACA |
| *Ins2* | GCTTCTTCTACACACCCATGTC | AGCACTGATCTACAATGCCAC |
| *Mafa* | AGGAGGAGGTCATCCGACTG | CTTCTCGCTCTCCAGAATGTG |
| *Pdx1* | CCCCAGTTTACAAGCTCGCT | CTCGGTTCCATTCGGGAAAGG |
| *Neurod1* | AGAAGAAGAGGAGGAGGAGG | TGACAGAGCCCAGATGTAG |
| *Nd4* | ACCATAGCCTTCTCACTATCAC | TGGAGTTGGAGTTTAGGGAAGT |
| *Nd6* | GGTTAGCATTAAAGCCTTCACC | CATCAACCAATCTCCCAAACCA |
| *As9* | GTCCGCTTTCGGGTTGTTAGA | CCTCCTTTCCCGTGAGGTA |
| *Cytb* | GCCACCTTGACCCGATTCT | TTGCTAGGGCCGCGATAAT |
| *Qcr1* | AGACCCAGGTCAGCATCTTG | GCCGATTCTTTGTTCCCTTGA |
| *Cytc* | CCAAATCTCCACGGTCTGTTC | ATCAGGGTATCCTCTCCCCAG |
| *Cox 1* | TCGGAGCCCCAGATATAGCA | TTTCCGGCTAGAGGTGGGTA |
| *Cox2* | CATGAGCAGTCCCCTCCCTA | TAACCCTGGTCGGTTTGATGT |
| *Cox3* | AAGGCCACCACACTCCTATT | ATCATGTGTTGGTACGAGGCT |
| *Cox4* | ATTGGCAAGAGAGCCATTTCTA | CACGCCGATCAGCGTAAGT |
| *Cox5b* | TTCAAGGTTACTTCGCGGAGT | CGGGACTAGATTAGGGTCTTCC |
| *Atpase6* | AGGATTCCCAATCGTTGTAGCC | CCTTTTGGTGTGTGGATTAGCA |
| *Atp5a1* | TCTCCATGCCTCTAACACTCG | CCAGGTCAACAGACGTGTCAG |
| *Atp5b* | GGTTCATCCTGCCAGAGACTA | AATCCCTCATCGAACTGGACG |
| *Atp5c1* | CCAGGAGACTGAAGTCCATCA | AGAACCTGTCCCATACACTCG |
| *18s rRNA* | CGCCGCTAGAGGTGCAATTC | CCAGTCGGCATCGTTTATGG |
| mtDNA |  |  |
| *16S rRNA* | CCGCAAGGGAAAGATGAAAGA | TCGTTTGGTTTCGGGGTTTC |

**Table S2. Antibodies for immunostaining and protein blotting**

| Antibody | company | Cat.NO |
| --- | --- | --- |
| Anti-AKT  Anti-pAKTs473 | Santa Cruz  Santa Cruz | sc-5298  sc-17753 |
| Anti-CREB | HuaBio | ET1601-15 |
| Anti-pCREB | HuaBio | ET7107-93 |
| Anti-CyclinD1 | HuaBio | ET1601-31 |
| Anti-CyclinB1 | HuaBio | ET1608-27 |
| Anti-Glucagon | HuaBio | ET1702-20 |
| Anti-Insulin | HuaBio | EM80714 |
| Anti-Ki67 | Abcame | ab15580 |
| Anti-Glut2 | Santa cruz | sc-518022 |
| Anti-PCNA | HuaBio | ET1605-38 |
| Anti-NeuroD | CST | #2833 |
| Anti-PDX1 | CST | #5679 |
| Anti-cl-caspase3 | CST | #9661 |
| Anti-syntaxin3 | HuaBio | ET7109-19 |
| Anti-SOD2 | HuaBio | ET1701-54 |
| Anti-Brdu | Abcame | (ab6326) |
| Anti-α-tubulin | HuaBio | M1501-1 |
